# Supplementary material for: DMRforPairs: identifying Differentially Methylated Regions between unique samples using array based methylation profiles
Source: BMC Bioinformatics. 2014 May 15;15:141. doi: 10.1186/1471-2105-15-141 (PMC4046028; doi:10.1186/1471-2105-15-141)
Supplement: Additional file 2 — DMRforPairs output for the comparison of A431-MCF7 and NA17018-NA17105. Please start from the HTML files in each folder. Available via the BMC Bioinformatics website. [file 1471-2105-15-141-S2.zip › 1394847754114233_MOESM2_ESM/A431_MCF7/figures/13645.pdf]

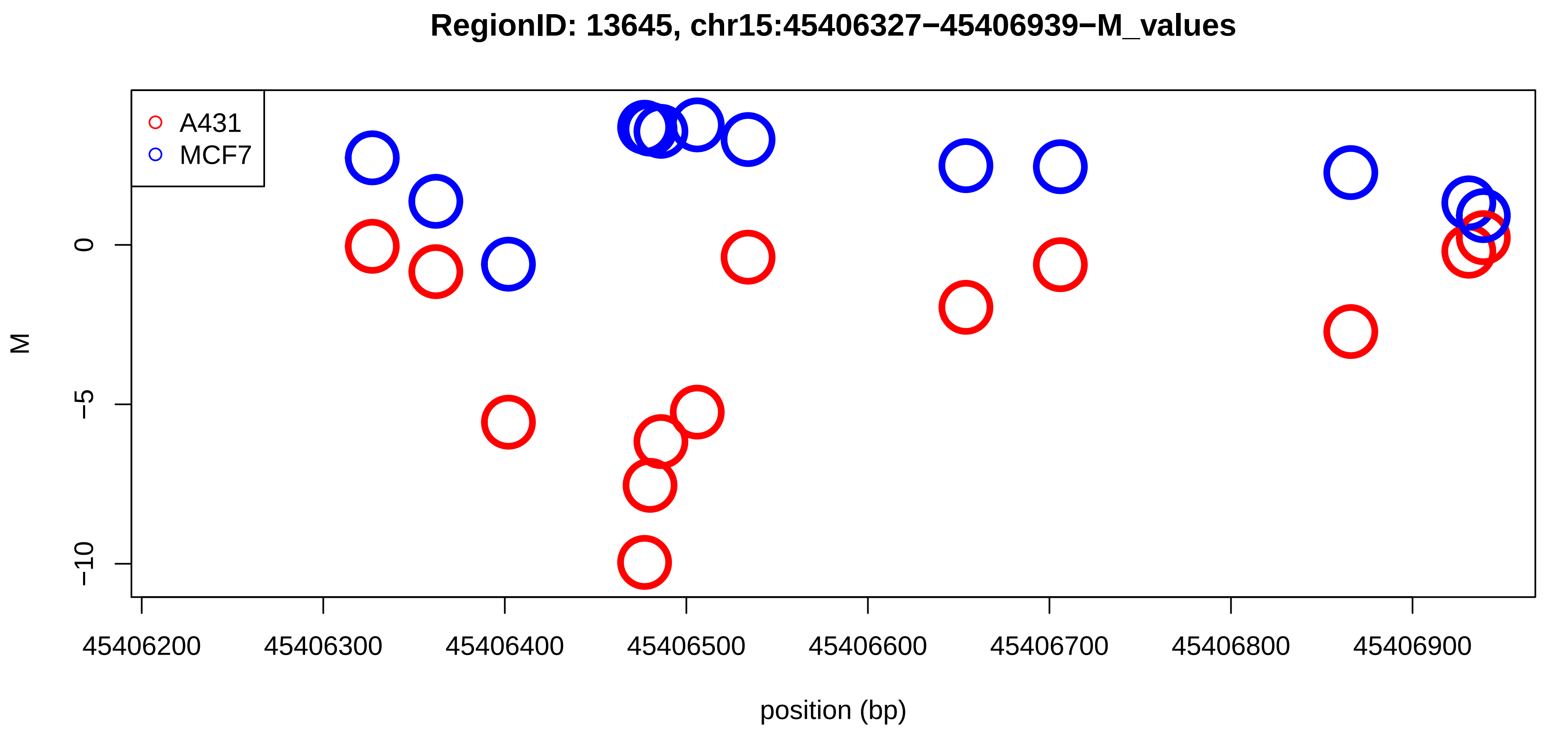
RegionID: 13645, chr15:45406327−45406939−M_values
A431
MCF7
M
0
−5
−10
45406200
45406300
45406400
45406500
45406600
45406700
45406800
45406900
position (bp)

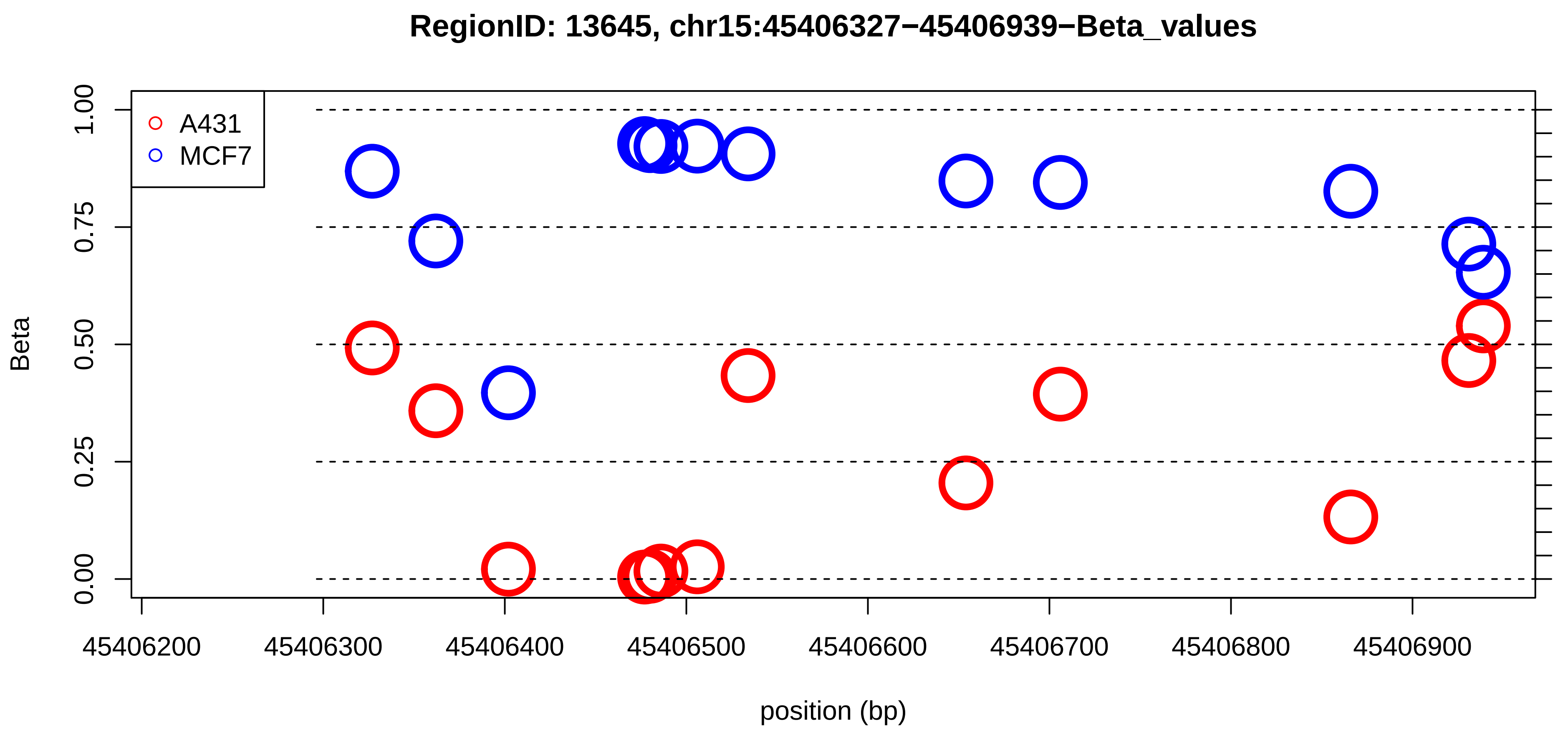
RegionID: 13645, chr15:45406327−45406939−Beta_values
A431
MCF7
Beta
1.00
0.75
0.50
0.25
0.00
45406200
45406300
45406400
45406500
45406600
45406700
45406800
45406900
position (bp)

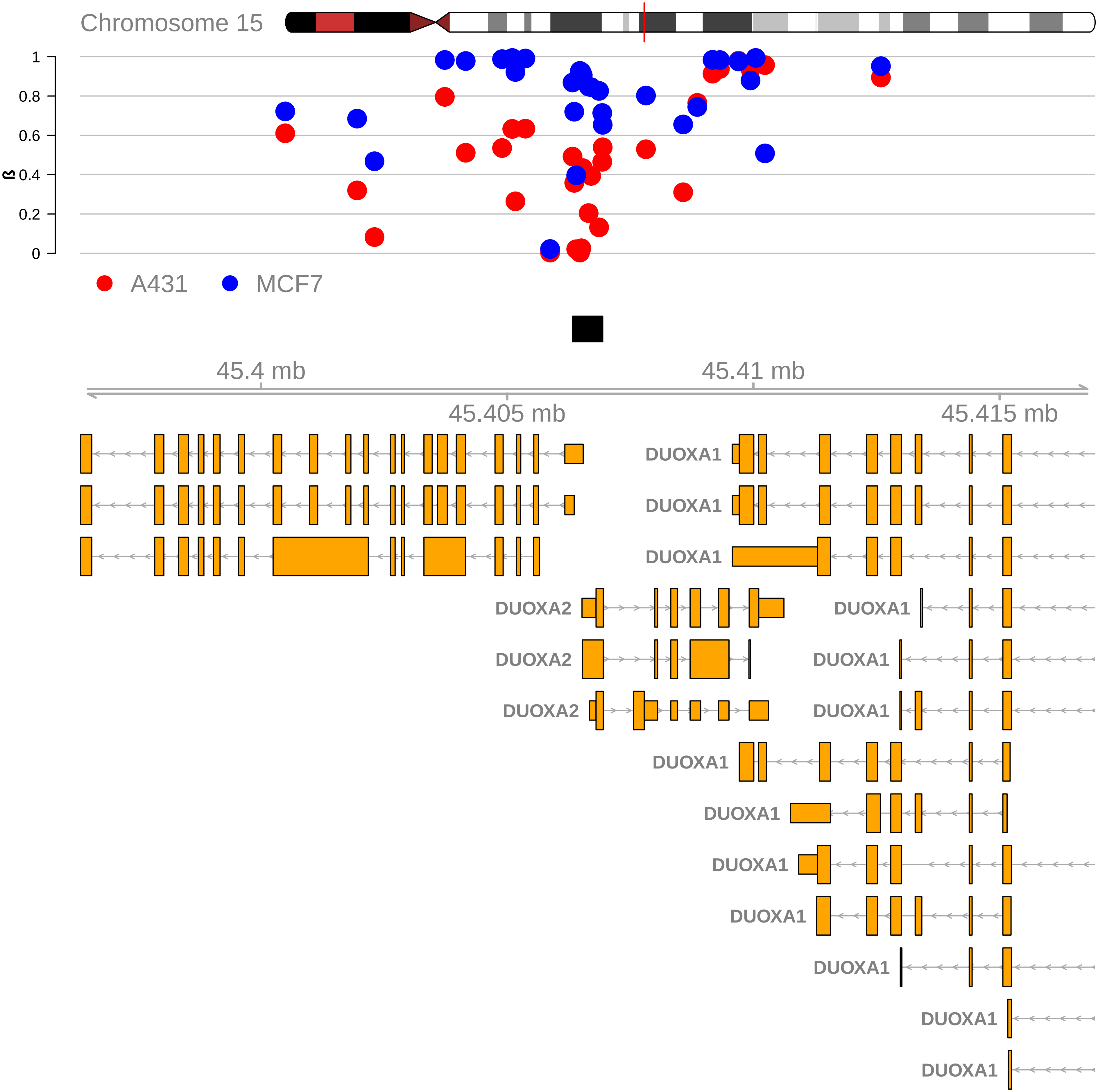
Chromosome 15
β
1
0.8
0.6
0.4
0.2
0
A431
MCF7
45.4 mb
45.41 mb
45.405 mb
45.415 mb
DUOXA1
DUOXA1
DUOXA1
DUOXA2
DUOXA1
DUOXA2
DUOXA1
DUOXA2
DUOXA1
DUOXA1
DUOXA1
DUOXA1
DUOXA1
DUOXA1
DUOXA1
DUOXA1
